# Longitudinal assessment of MPXV-specific IgG avidity in patients with Mpox infection

**DOI:** 10.3389/fcimb.2026.1882915

**Published:** 2026-08-24

**Authors:** Luigi Rosa, Aurora Bettini, Giulia Matusali, Licia Bordi, Andrea Antinori, Valentina Mazzotta, Fabrizio Maggi, Francesca Colavita

**Affiliations:** 1 National Institute for Infectious Diseases Lazzaro Spallanzani-IRCCS, Rome, Italy; 2 Department of Biology and Biotechnology “Charles Darwin”, Sapienza University of Rome, Rome, Italy; 3 European Union Public Health Reference Laboratories for Emerging, Rodent-borne and Zoonotic Viral Pathogens (EURL-PH-ERZV), Rome, Italy

**Keywords:** avidity, IgG, mpox, MVA-BN vaccination, serology

## Abstract

**Introduction:**

Monkeypox virus (MPXV) infection induces robust humoral immune responses; however, the kinetics of IgG avidity maturation following natural infection remain poorly characterized. Since IgG avidity reflects antibody affinity maturation over time, its evaluation may provide additional information for the interpretation of MPXV serology and infection staging. This study longitudinally assessed MPXV-specific IgG avidity in patients with confirmed mpox infection up to one year after symptom onset.

**Methods:**

Serum samples from 16 PCR-confirmed mpox patients (clade IIb) were collected within 30 days from symptom onset (T1) and at 3 (T2), 6 (T3), and 12 (T4) months of follow-up, each comprising 13 samples. Anti-MPXV (clade IIb) IgG titers and avidity indices (AI) were evaluated using an in-house indirect immunofluorescence assay (iIFA) performed with and without 4.5 M urea treatment. Neutralizing antibodies (nAbs) were measured by plaque reduction neutralization test (PRNT_50_). ROC curve analysis was performed to assess the ability of AI to discriminate early from late infection time points.

**Results:**

All patients developed detectable anti-MPXV IgG within the first month after symptom onset, followed by a progressive decline in antibody titers over time, with average reductions of 1.5-, 2.9-, and 5.8-fold at T2, T3, and T4, respectively. In contrast, IgG avidity progressively increased during follow-up. Mean AI values increased from 0.132 at T1 to 0.619, 0.653, and 0.689 at T2, T3, and T4, respectively. ROC analysis identified an AI cutoff of 0.25 capable of discriminating samples collected within 30 days from those collected at later time points with high accuracy. An inverse correlation between AI and nAbs titers was observed (Spearman’s r = −0.5131, p < 0.0001), suggesting distinct temporal dynamics between antibody maturation and neutralizing activity.

**Discussion:**

These findings provide one of the first longitudinal descriptions of IgG avidity maturation following natural MPXV infection and support the potential utility of avidity assessment as a complementary serological parameter for distinguishing recent from past infection. Further studies in larger and more diverse cohorts are needed to validate these observations and better define the clinical applicability of MPXV IgG avidity testing.

## 1 Introduction

Mpox, caused by the monkeypox virus (MPXV), is a zoonotic infection due to an Orthopoxvirus that has gained global relevance following the multicountry outbreak of 2022. The disease typically presents with fever, lymphadenopathy, and a vesiculopustular rash affecting skin and mucosal surfaces, with severe manifestations more frequently observed in immunocompromised individuals (Adler et al., 2022). In routine clinical practice, diagnosis primarily relies on nucleic acid detection from skin or mucosal swabs, while serology is increasingly used in seroepidemiological studies, in monitoring post-vaccination immunity, and in characterizing the humoral response following natural infection (Grossegesse et al., 2023).

Recent studies have described the kinetics of the humoral immune response to MPXV, demonstrating early induction of IgM and IgG antibodies followed by the development of neutralizing antibodies (nAbs), with variable magnitude and persistence (Yang et al., 2024; Raccagni et al., 2024). In parallel, the global implementation of the non-replicating Modified Vaccinia Ankara-Bavarian Nordic (MVA-BN) vaccine has enabled systematic evaluation of vaccine-induced immunity. Although most vaccine recipients develop detectable IgG against MPXV/Vaccinia virus antigens, antibody levels decline over time and may become undetectable within one year after the primary two-dose regimen (Mazzotta et al., 2024; van Leeuwen et al., 2024). Notably, Oom et al. (2025) showed that individuals without prior MPXV exposure develop predominantly low-avidity IgG after MVA-BN vaccination, whereas subjects with historical smallpox vaccination mount more durable and high-avidity responses. These findings highlight the importance of assessing not only the magnitude but also the quality of the humoral response.In this context, IgG avidity represents a relevant serological parameter. Avidity reflects the overall binding strength of a polyclonal antibody population to its antigen, integrating the intrinsic affinity of individual binding sites and the multivalent interactions of IgG molecules (Li et al., 2025). Following primary infection, IgG antibodies initially exhibit low avidity, which progressively increases as a result of affinity maturation processes occurring in germinal centers, typically stabilizing over weeks to months (Garcia et al., 2022). Accordingly, higher avidity values generally indicate a longer time since infection. IgG avidity is commonly assessed by ELISA or indirect immunofluorescence assay (iIFA) incorporating a chaotropic agent (e.g., urea) to dissociate low-affinity interactions, with results expressed as an avidity index or titer ratio (Pereira et al., 2001; Nurmi et al., 2021).

IgG avidity testing has been extensively validated in multiple viral infections and related diseases. It is routinely used to distinguish recent from past infection in rubella, particularly in early pregnancy to exclude first-trimester infection associated with a high risk of congenital defects (Agbede et al., 2013). Similarly, anti-cytomegalovirus (CMV) IgG avidity measurement is a sensitive and specific tool for identifying recent primary infection and assessing the risk of vertical transmission (Prince and Lapé-Nixon, 2014). Avidity testing is also informative in parvovirus B19 and herpesvirus-7 infections, aiding in the distinction between primary infection, reactivation, or cross-reactive serological patterns (Pereira et al., 2001; Prince and Lapé-Nixon, 2014; Magalhães et al., 2011). In dengue virus infection, combined interpretation of the IgM/IgG ratio and IgG avidity enables reliable discrimination between primary and secondary infections, even when samples are collected ≥30 days after symptom onset (Prince et al., 2011). In Zika virus infection, avidity testing provides essential temporal information in the presence of flavivirus cross-reactivity and supports clinical management during pregnancy (Amaro et al., 2019; Bouthry et al., 2020). More recently, studies on SARS-CoV-2 have shown that, despite declining IgG titers, antibody avidity increases over time after infection or vaccination, contributing to improved estimation of time since exposure (Garcia et al., 2022).

Importantly, differences in avidity maturation may help distinguish infection-induced from vaccine-induced responses. In SARS-CoV-2 infection, Struck et al. (2021) demonstrated that natural infection may result in incomplete avidity maturation, whereas mRNA vaccination induces rapid development of high-avidity IgG, particularly against the receptor-binding domain. These differences have been associated with variations in antibody functionality, durability of humoral immunity, and interpretation of serological protection. Conversely, atypical avidity profiles—such as high-avidity binding to linear epitopes combined with low overall functional avidity—have been described in Borna disease virus (BDV) infection, highlighting the complexity of humoral responses beyond IgG titers alone (Billich et al., 2002).

Despite its established role in other viral infections, IgG avidity in MPXV infection remains poorly characterized. In particular, data on the kinetics of avidity maturation following natural infection and on its relationship with neutralizing activity are still limited.

The present study aimed to longitudinally characterize the humoral response to MPXV infection by assessing anti-MPXV IgG levels and IgG avidity in serum samples collected up to one year after symptom onset. Using an iIFA-based approach, we compared IgG titers under standard conditions and after urea treatment to evaluate avidity maturation over time. In addition, IgG avidity indices were correlated with MPXV-specific nAbs to explore the relationship between antibody maturation and functional neutralization during follow-up.

## 2 Materials and methods

### 2.1 Study samples

A total of 52 serum samples, collected from 16 MPXV clade IIb-infected patients between 2022 and 2023 were analyzed.

The study was conducted at the Lazzaro Spallanzani National Institute for Infectious Diseases IRCCS (Rome, Italy). The study was approved by the Ethics Committee of the Lazzaro Spallanzani Institute (approval number 40z, Register of Non-COVID Trials, approval date: 8 September 2022). All participants provided written informed consent. The samples were stratified according to days from symptom onset (dso): <30 days (T1), 3 months (T2), 6 months (T3), and 1 year (T4), each comprising 13 samples. The distribution of sampling times showed a median of 18 dso (interquartile range [IQR]: 11–18.5) for T1, 109 dso (IQR: 100–122.5) for T2, 228 dso (IQR: 220–250) for T3, and 381 dso (IQR: 363–412.5) for T4.

### 2.2 Serological diagnostic

Specific IgG antibodies were detected by an iIFA as previously described (Colavita et al., 2019), using slides prepared in-house with Vero E6 cells. Briefly, slides were prepared at the National Institute for Infectious Diseases “Lazzaro Spallanzani” Biosafety Level 3 (BSL-3) laboratory in Rome using Vero E6 cells infected with an MPXV isolate obtained from a skin lesion of a patient during the 2022 outbreak (GenBank: ON745215.1). At 24 h post-infection, cells were trypsinized, mixed 1:1 with uninfected Vero E6 cells, washed twice in Dulbecco’s phosphate-buffered saline (PBS; Sigma-Aldrich, St. Louis, MO, USA), and fixed in acetone for 30 min at −20 °C.

Serum samples were initially diluted 1:20 in PBS and then serially diluted up to 1:10,240 to determine endpoint titers. After incubation for 1 h at 37 °C, slides were washed and incubated for 1 h at 37 °C with a fluorescein isothiocyanate (FITC)-conjugated anti-human IgG antibody and Evans Blue counterstain (Euroimmun, Lübeck, Germany). Fluorescence was evaluated using a Nikon Eclipse E200 microscope. Positive and negative controls were included in each run. Assay setup was performed using 30 serum samples from healthy donors born after the 1970s, to minimize cross-reactivity due to historical smallpox vaccination. In addition, sera from individuals with prior smallpox vaccination were included as controls based on previously reported cross-reactive humoral responses (Zaeck et al., 2023). All samples analyzed in this study were obtained from PCR-confirmed mpox patients.

**Figure 1:**
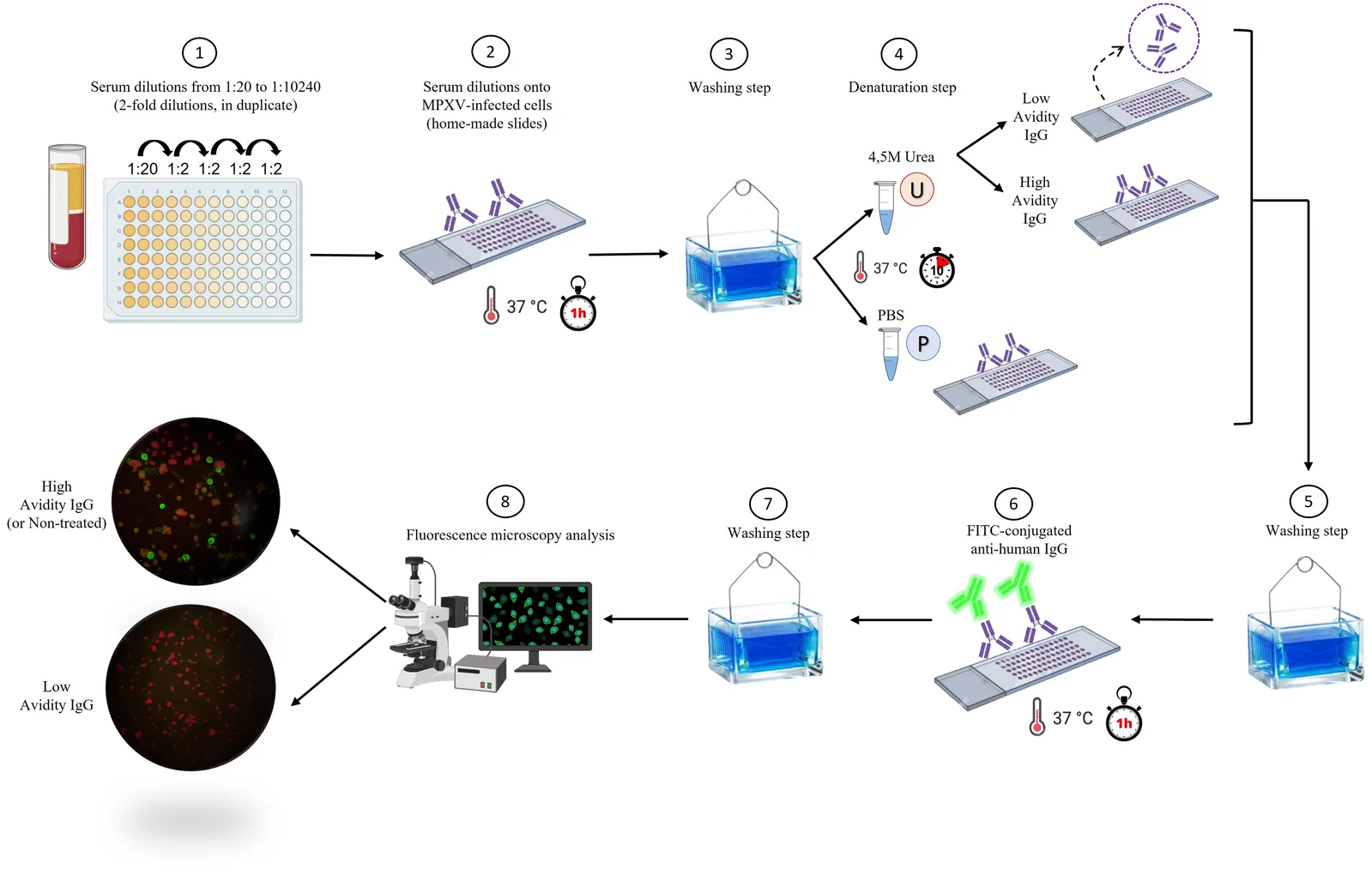
Schematic representation of the IgG avidity assay workflow. (1) Preparation of serum dilutions from 1:20 to 1:10240 (2-fold dilutions, in duplicate) in PBS; (2) Serum dilutions spotted onto home-made slides prepared with clade IIb MPXV-infected cells and incubation at 37 °C for 1 hour; (3) three washes for 10 min/each in PBS supplemented with 0.05% Tween-20;(4) Sample treatment with 4.5 M Urea or PBS (untreated control), both incubated at 37 °C for 10 min.; (5) three washes for 10 min/each in PBS supplemented with 0.05% Tween-20; (6) FITC-conjugated anti-human IgG staining and incubation at 37 °C for 1 hour; (7) three washes for 10 min/each in PBS supplemented with 0.05% Tween-20; (8) Fluorescence microscopy analysis for IgG titer estimation through end-point dilution method; avidity index calculated comparing urea treatment and not treated samples (high avidity presence of fluorescence; low avidity, absence of fluorescence).

IgG avidity was assessed using the same iIFA platform with an endpoint dilution approach as shown in **Figure 1**. Urea (Sigma LifeScience, Whitby, ON, Canada) was used as a chaotropic agent to disrupt antigen–antibody interactions and assess antibody binding strength under denaturing conditions. For assay optimization, different urea concentrations and treatment protocols were evaluated. Urea treatment was performed for 10 min at 37 °C using two alternative protocols: (i) direct application of urea at concentrations of 3, 4.5, or 6 M after serum incubation, and (ii) washing with PBS containing urea at final concentrations of 2.5 or 4.5 M before serum incubation. Optimization experiments were conducted using two control serum samples collected at different stages of infection: one during the acute phase, within 6 days of symptom onset, and the other 12 months after infection. Based on the comparative performance of the tested conditions, direct application of 4.5 M urea for 10 min at 37 °C, following serum incubation for 30 min at 37 °C, was selected as the optimal condition for the avidity assay.

After urea treatment, slides were washed three times for 10 min in PBS supplemented with 0.05% Tween-20, followed by incubation with FITC-conjugated anti-human IgG (Euroimmun, Lübeck, Germany) and fluorescence microscopy analysis (**Figure 1**). All experiments were performed in parallel under non-denaturing conditions (non-treated, nt) to obtain reference IgG titers for avidity index (AI) calculation.

The AI was calculated as the ratio between the IgG titer obtained under nt conditions and that measured after urea treatment. A ≥4-fold reduction in antibody titer after urea treatment was considered indicative of low-avidity antibodies.

### 2.3 Neutralization assays

Clade IIb MPXV-specific neutralizing antibodies (nAbs) were quantified using a 50% plaque reduction neutralization test (PRNT_50_) starting from a 1:10 serum dilution. Serum samples were heat-inactivated at 56 °C for 30 min and titrated in duplicate across four serial four-fold dilutions. Each serum dilution was mixed 1:1 with a viral solution containing 100 TCID_50_ of the MPXV isolate (GenBank: ON745215.1, derived from the clinical sample) and incubated for 2 h at 37 °C. Sub-confluent Vero E6 monolayers were then infected with the virus–serum mixtures and maintained at 37 °C in 5% CO_2_.

After 5 days of incubation, supernatants were gently removed and cells were fixed and stained with a crystal violet solution (Diapath S.p.A., Martinengo, BG, Italy) containing 10% formaldehyde (Sigma–Aldrich, St. Louis, MO, USA). Following a 30-min incubation, the fixative was discarded and the monolayers were washed with 1× PBS. Plaques were enumerated using the Cytation 5 imaging reader (BioTek, Winooski, VT, USA). Neutralizing titers were calculated as the reciprocal of the highest serum dilution that resulted in ≥50% reduction in plaque count compared with virus-only control wells.

Each assay included a serum control (1:10 dilution of each sample without virus in duplicate), a cell control (uninfected Vero E6 monolayers, in six replicate), and a virus control (100 TCID_50_ MPXV in octuplicate).

### 2.4 Statistical analysis

All statistical analyses were performed using GraphPad Prism 10 XML ProjecT (San Diego, CA, USA). Patient characteristics were summarized as median and IQR for continuous and percentage for categorical variables. Geometric mean titer (GMT) and 95% confidence interval (CI) were calculated to express the antibody levels as well as AI values. Statistical significance was assessed using the non-parametric Kruskal–Wallis test for independent samples or, when applicable, the paired non-parametric Wilcoxon test. Receiver operating characteristic (ROC) curve analysis was performed using MedCalc software (Ostend, Belgium) to determine the optimal AI cutoff for discriminating samples collected at <30 days from those collected at ≥3 months after symptom onset. The optimal cutoff value was identified using the Youden index, which maximizes the sum of sensitivity and specificity. Correlations between AI and nAbs titer were assessed with Spearman’s rank correlation. Statistical significance was defined as p<0.05.

## 3 Results

### 3.1 Demographics

The main characteristics of the study population are summarized in **Table 1**. Sixteen mpox patients were included in the study. All were self-reported men who have sex with men (MSM), with a median age of 39 years (IQR: 36.25–43.75). Five (31.25%) were people living with HIV (PLWH), with a median CD4+ T-cell count of 586 cells/μL (IQR: 521.5–893.5) recorded within the previous three months. All PLWH included in the study were on stable antiretroviral therapy at the time of mpox diagnosis. Three of 16 patients (18.75%) received antiviral therapy for MPXV infection (two with tecovirimat and one with cidofovir). None of the patients had received the smallpox vaccination during childhood.

**Table 1:** Main demographic characteristics of mpox patients.

Demographic mpox patients	
Male (n, %)	16 (100)
Age (Median, IQR)	39 [36.25-43.75]
PLWH ^1^ (n, %)	5 (31.25)
CD4 count (cells/μL) (Median, IQR)	586 [521.5-893.5]
Antiviral therapy (n, %)	3 (18.75)
No antiviral therapy (n, %)	13 (81.25)

^1^PLWH, people living with HIV; IQR, interquartile range.

### 3.2 Kinetics of anti-MPXV IgG titers with and without urea treatment

**Figure 2:**
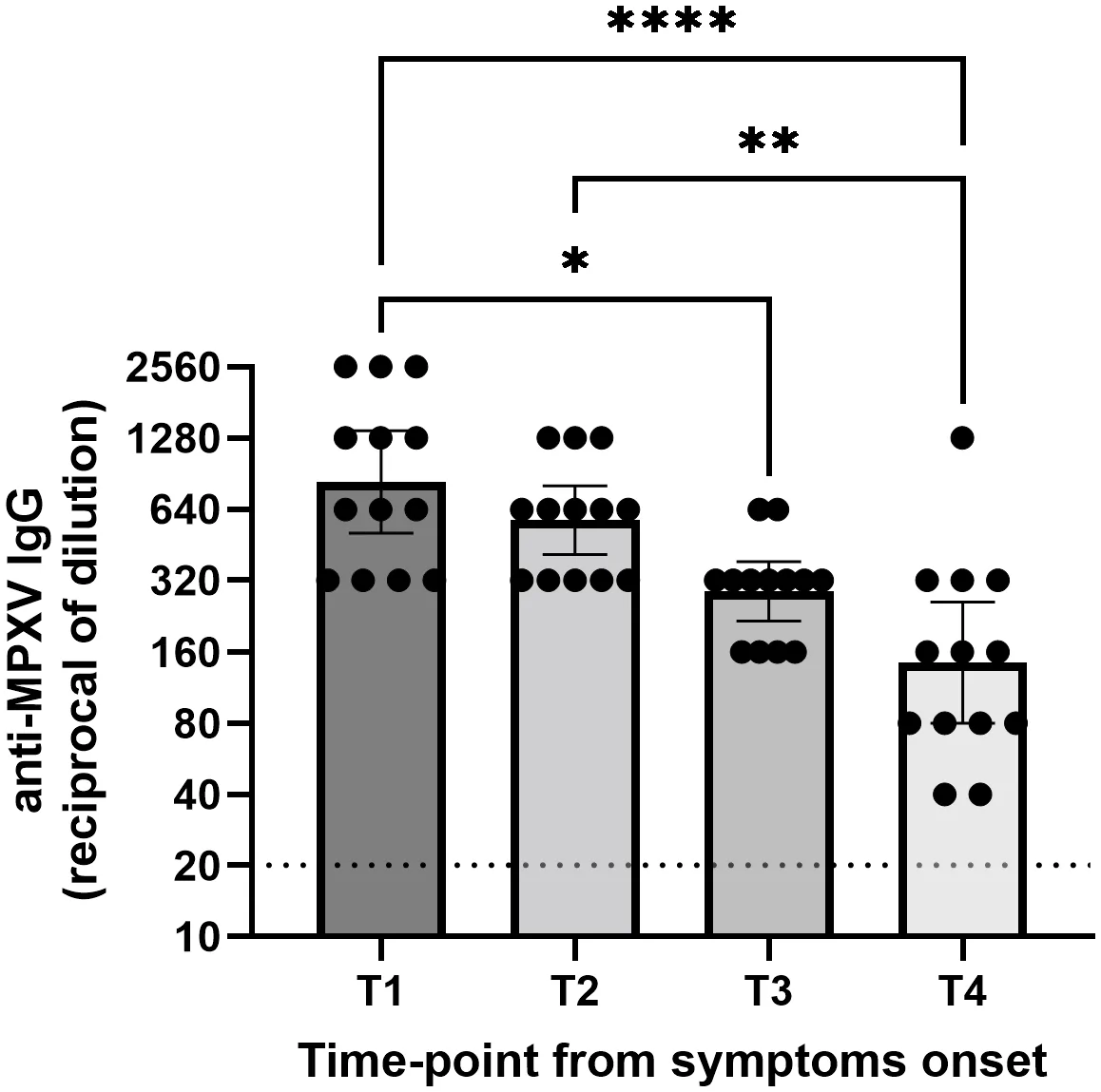
Titers of anti-MPXV IgG measured by indirect immunofluorescence assay (iIFA) in 16 patients at different time points according to days from symptom onset (dso): <30 days (T1), 3 months (T2), 6 months (T3), and 1 year (T4), each comprising 13 samples. Dot black line indicates the limit of the assay detection (starting dilution of 1:20). Data are represented as geometric mean titer (GMT) and 95% confidence interval (CI). Statistical significance was defined as p<0.05. Statistical significance is indicated as follows: *: p < 0.05; **: p < 0.01; ****: p < 0.0001.

Within the first month from symptom onset, all patients developed high anti-MPXV IgG levels. Antibody titers gradually declined over time, with an average of 1.5-, 2.9-, and 5.8-fold reduction at T2, T3, and T4, respectively, compared to T1; however, all individuals remained IgG-positive at the one-year follow-up. Consistent with this trend, anti-MPXV IgG titers measured under non-denaturing conditions displayed a clear temporal trajectory (**Figure 2**). IgG levels were highest at T1, with a GMT of 835.5 and a 95% CI of 506.9-1377, indicating inter-individual variability in the short-term immune response. At T2, titers decreased to a GMT of 575.3 (95% CI: 411.4-804.5), and further declined at T3, reaching a GMT of 287.6 (95% CI: 215.6-383.8). The lowest titers were observed at T4, with a GMT of 143.8 (95% CI: 79.8-259.1).

**Figure 2** illustrates a clear downward trajectory, with statistically significant differences between early and late time points. Specifically, anti-MPXV IgG titers at T4 were significantly lower than at T1 (p<0.0001), and reductions were observed in the comparisons T1 vs T3 (p <0.05) and T2 vs T4 (p<0.01).

**Figure 3:**
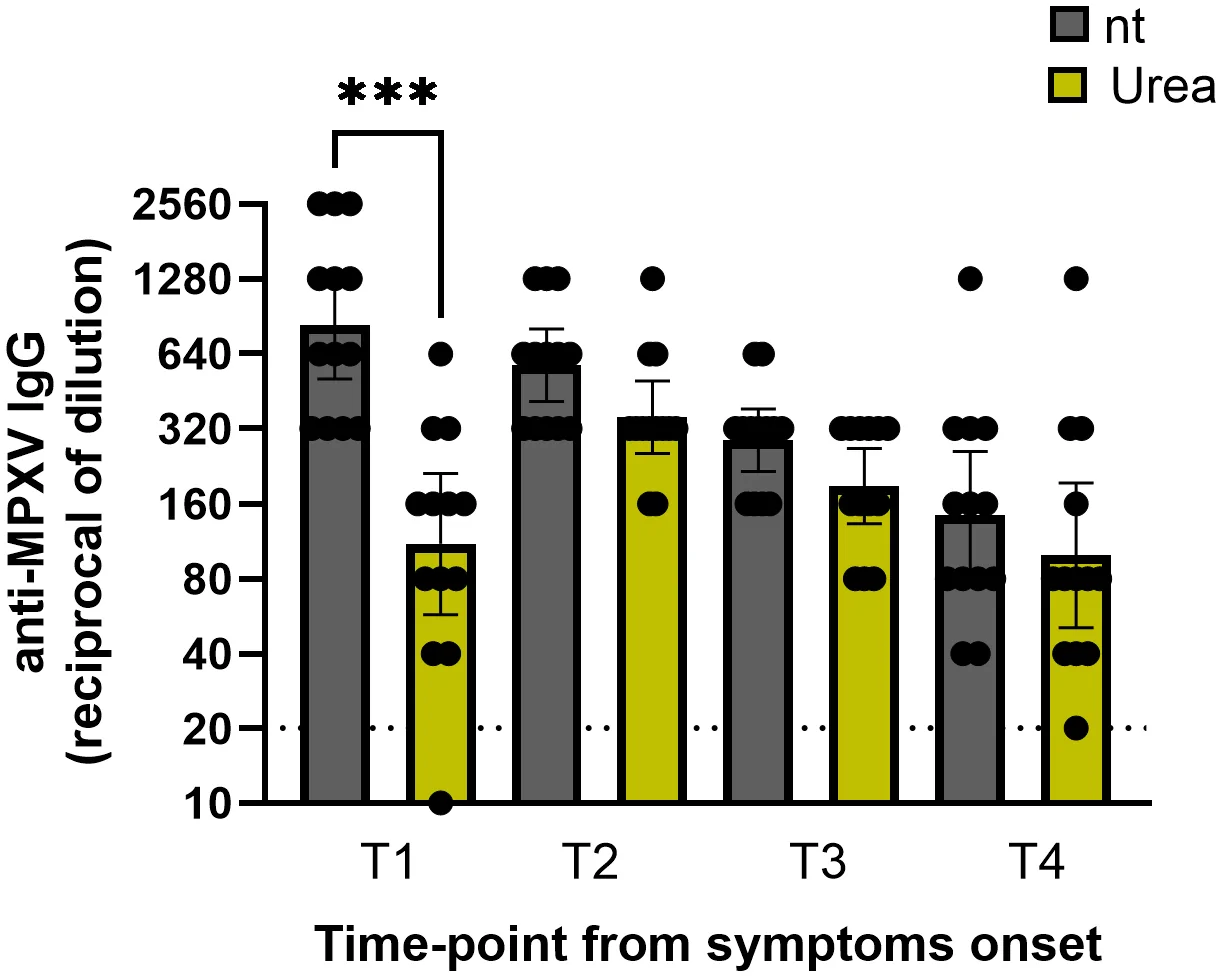
Titers of anti-MPXV IgG measured by indirect immunofluorescence assay (iIFA) with or without (nt) 4.5 M urea treatment in 16 patients at different time points according to days since symptom onset (dso): <30 days (T1), 3 months (T2), 6 months (T3), and 1 year (T4), each comprising 13 samples. Dot black line indicates the limit of the assay detection (starting dilution of 1:20). Data are represented as geometric mean titer (GMT) and 95% confidence interval (CI). Statistical significance was defined as p<0.05. Statistical significance is indicated as follows: ***: p < 0.001.

A direct comparison of nt and urea-treated iIFA titers (**Figure 3**) demonstrated the effect of denaturation on MPXV-specific IgG binding. At T1, the reduction in titers after urea treatment was statistically significant (p<0.001), consistent with the presence of predominantly low-avidity antibodies in the early phase of infection. In contrast, at T2, T3, and T4, the discrepancy between nt and urea-treated titers progressively decreased, reflecting increased resistance of antigen–antibody interactions to chaotropic disruption. By T4, several samples (7/13) exhibited same titers in both conditions, supporting the notion of advanced affinity maturation.

**Figure 4:**
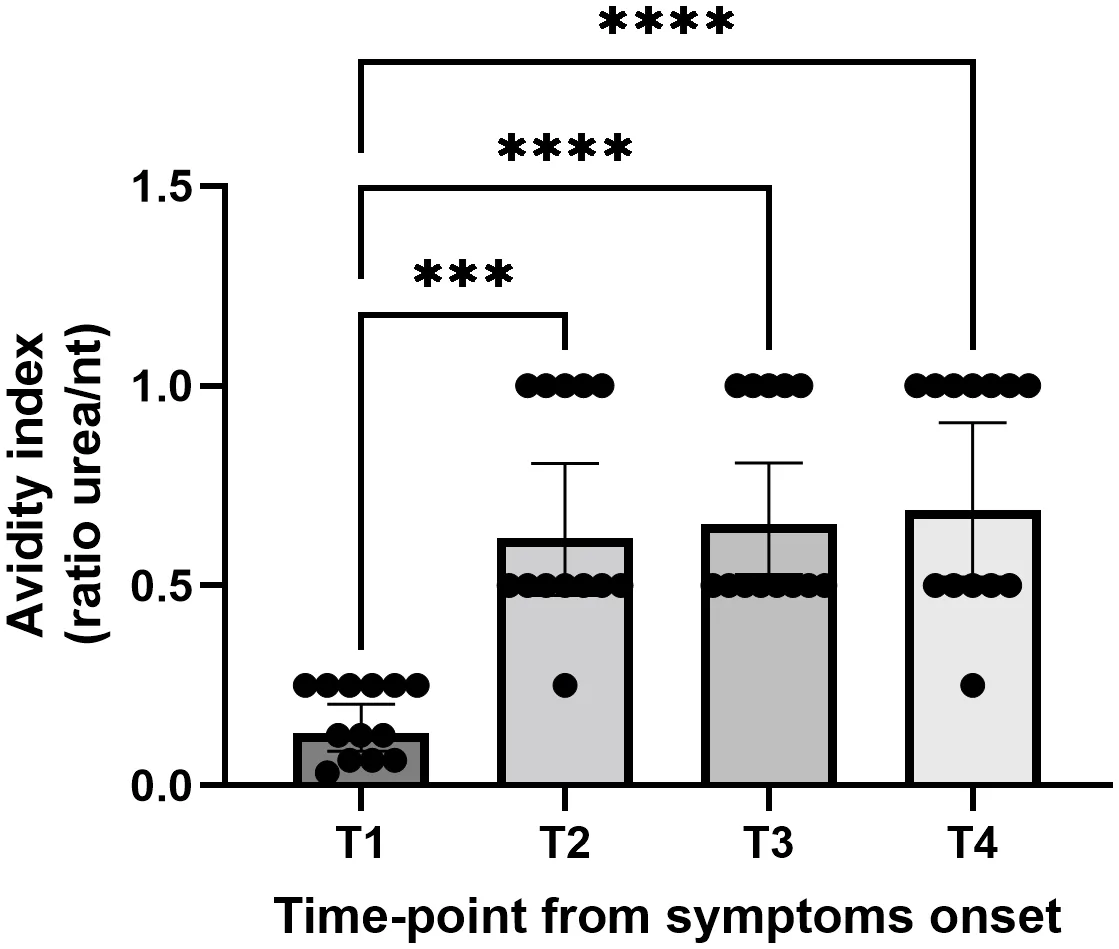
Avidity index of anti-MPXV IgG measured by indirect immunofluorescence assay (iIFA) performed with 4.5 M urea in 16 patients at different time points according to days since symptom onset (dso): <30 days (T1), 3 months (T2), 6 months (T3), and 1 year (T4), each comprising 13 samples. The avidity index was calculated as the ratio between the IgG titer obtained under non-treated (nt) conditions and the corresponding titer measured after urea treatment. Data are represented as geometric mean titers (GMT) and 95% confidence interval (CI). Statistical significance was defined as p<0.05. Statistical significance is indicated as follows: ***: p < 0.001; ****: p < 0.0001.

When antibody binding was evaluated under denaturing conditions (4.5 M urea), a clear temporal pattern of IgG avidity maturation emerged (**Figure 4**). At T1, samples exhibited very low avidity, with AI values for all patients (n=13, 100%) clustering below the 0.3 value (average AI: 0.132; 95% CI: 0.085-0.204). A significant increase was observed at T2 as compared to T1 (p<0.001), with average AI of 0.619 (95% CI: 0.476-0.806); at this time point, 1/13 patients had an AI < 0.3, 7/13 displayed values of 0.5, and 5/13 reached an index of 1.0. This increase remained significant both at T3 and T4 versus T1 (p<0.0001), with average AI of 0.653 (95% CI: 0.529–0.807) at T3 and 0.689 (95% CI: 0.522–0.909) at T4. More in detail, at T3, 8/13 patients showed an AI of 0.5 and 5/13 reached an index of 1.0, whereas at T4, 1/13 had an avidity index < 0.3, 5/13 had values around 0.5, and 7/13 reached 1.0. Despite the small sample size, no significant differences were observed between PLWH and PWoH in either AI values or the kinetics of avidity maturation.

**Figure 5:**
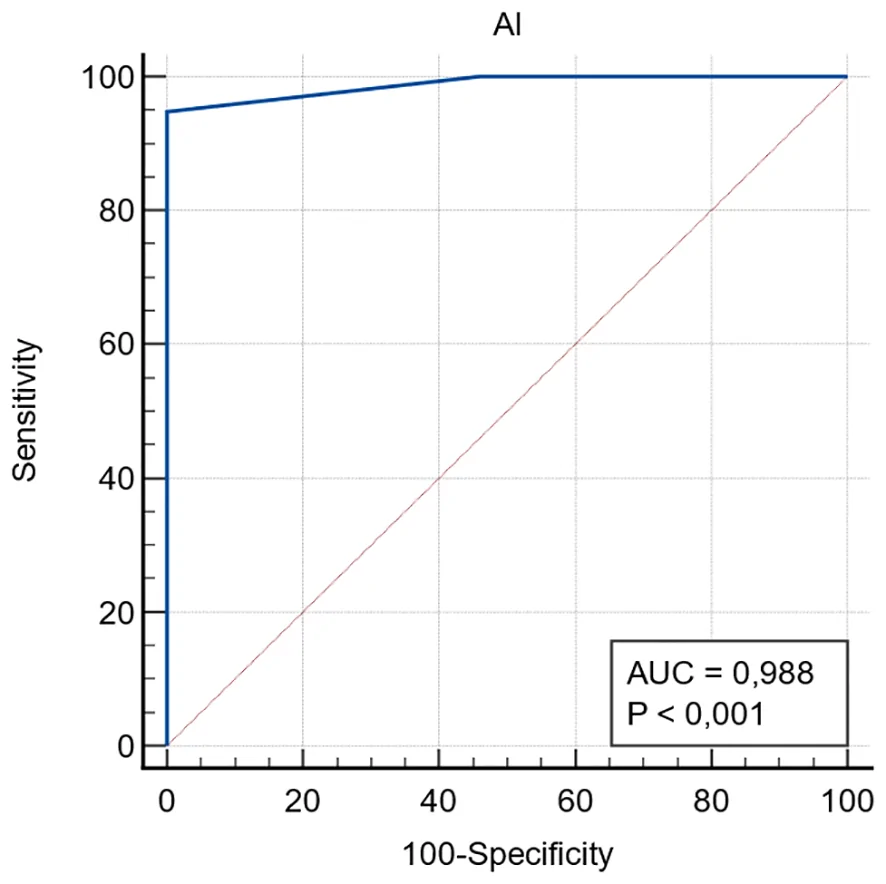
Receiver operating characteristic (ROC) curve of MPXV IgG avidity index (AI) measured by indirect immunofluorescence assay (iIFA) performed with 4.5 M urea. Statistical significance was defined as p<0.05.

ROC curve analysis was performed to determine the optimal cutoff value of the IgG AI for discriminating recent from past MPXV infection. The AI showed excellent discriminatory performance, with an area under the curve (AUC) of 0.988 (95% CI: 0.910–1.000, p<0.0001) (**Figure 5**). The optimal cutoff was AI >0.25, which yielded a sensitivity of 94.87% and a specificity of 100% for identifying past MPXV infection (samples collected at ≥3 months after symptom onset). Conversely, AI values ≤0.25 were indicative of recent infection (samples collected at <30 days after symptom onset).

### 3.3 Correlation between IgG avidity and neutralizing antibody

**Figure 6:**
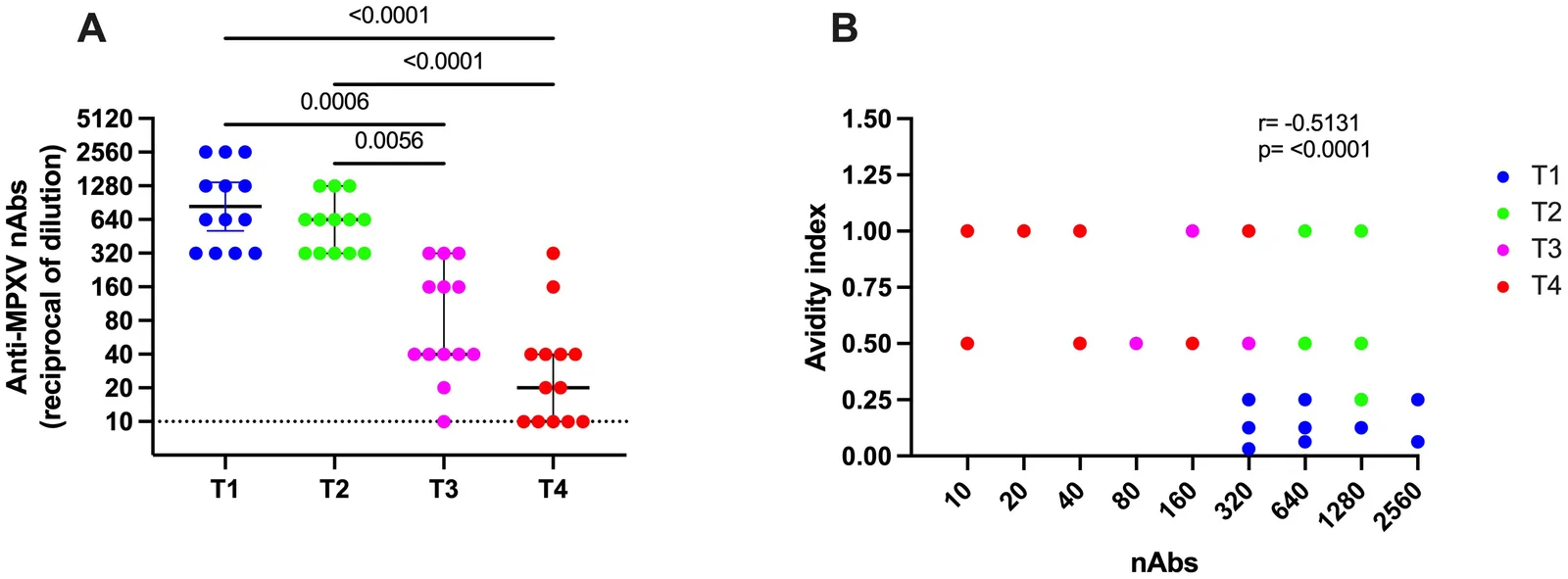
**(A)** Longitudinal evaluation of MPXV-specific neutralizing antibody (nAb) titers, expressed as 50% plaque reduction neutralization test (PRNT_50_) values, in serum samples collected within 30 days (T1), and at 3 (T2), 6 (T3), and 12 (T4) months from symptom onset. Each dot represents an individual serum sample. Horizontal bars indicate the geometric mean titer (GMT) with 95% confidence intervals (CI). The dot black lines indicate the limit of the assay detection (starting dilution of 1:10). Statistical comparisons were performed using the using the Kruskal-Wallis test followed by Dunn’s multiple-comparison test. **(B)** Scatter plot showing the relationship between the avidity index (ratio of urea-treated to untreated iIFA titers) and nAbs expressed as PRNT_50_ in samples collected at T1, T2, T3 and T4 from symptom onset. Statistical significance was defined as p<0.05.

Neutralizing antibody (nAb) titers were evaluated longitudinally by PRNT_50_ at all time points (**Figure 6A**). The GMT of nAbs was 835.5 (95% CI: 506.9–1377) at T1, 575.3 (95% CI: 411.4–804.5) at T2, 75.8 (95% CI: 37.9–151.8) at T3, and 27.54 (95% CI: 14.0–54.1) at T4. Compared with earlier follow-up time points, nAb titers showed a progressive decline over time, reaching significantly lower levels at T4 than at T1 and T2 (p < 0.0001 for both comparisons). In addition, nAb titers were significantly lower at T3 than at T2 (p = 0.0056) and at T2 than at T1 (p = 0.0006).

To evaluate the relationship between antibody maturation and functional neutralization capacity, correlation analysis was performed including samples from all-time points (T1–T4). Spearman’s rank correlation demonstrated a statistically significant modest inverse association between IgG AI and nAbs titers measured by PRNT_50_ (r = −0.5131, p < 0.0001) (**Figure 6B**).

Higher nAbs titers were generally observed in samples with lower AI, particularly during early infection, indicating a time-dependent relationship between functional neutralization and affinity maturation.

## 4 Discussion

Despite the increasing availability of serological assays for MPXV, their clinical interpretation remains challenging. Current serological tests primarily detect binding antibodies against Orthopoxvirus antigens and often suffer from cross-reactivity with other poxviruses or historical smallpox vaccination, limiting their ability to define the timing and nature of exposure. Moreover, conventional serology cannot reliably distinguish recent infection from past exposure or vaccination-induced immunity (Meyer et al., 2020). In fact, although pathogen-specific IgM antibodies are widely used as markers of recent infection, their diagnostic value may have important limitations. IgM responses may persist for months after primary infection, can be absent or transient during secondary infections or in case of immunosuppression, and are often affected by cross-reactivity among closely related pathogens, leading to reduced specificity. IgG avidity reflects the maturation of the humoral immune response and is less influenced by these factors, making it a useful complementary additional serological marker to refine infection staging, particularly in individuals with unclear exposure history or in epidemiological investigations aiming to distinguish recent from past infection. In this context, the characterization of IgG avidity maturation is needed to assess whether avidity testing can effectively contribute to solving current limitations of MPXV serology. The present study provides one of the first longitudinal evaluations of IgG avidity maturation following confirmed mpox infection, offering new insights into the quality and durability of the humoral response up to one year after symptom onset. Consistent with observation in larger study cohort (Mazzotta et al., 2025), all patients included in this study developed high anti-MPXV IgG titers within the first month after symptom onset. Although antibody titers gradually declined over time, all individuals remained IgG positive at the one year follow up, thus confirming the persistence of binding antibodies long after viral clearance.

During the early phase of infection, within 30 days from symptom onset, IgG responses were uniformly characterized by low avidity, consistent with the immunological expectation that initial antibody responses are predominantly composed of low-affinity antibodies prior to immune maturation. From three months after disease onset onward, we observed a clear and progressive increase in IgG avidity, with many patients reaching maximal avidity values between 6 and 12 months. Consistently, although these results should be confirmed on larger cohorts, ROC curve analysis identified an AI cutoff of 0.25 able to discriminate with high accuracy samples collected within 30 days from those collected at later time points, supporting the use of avidity as a marker of infection timing. Overall, we observed that the IgG AI can represent a useful serological parameter for distinguishing recent from past MPXV infection during follow-up. These observations are consistent with evidence from other viral infections, in which avidity analysis has been validated and, in some cases, is routinely used in clinical diagnostics, particularly in the context of pregnancy management (Agbede et al., 2013; Prince and Lapé-Nixon, 2014).

Interestingly, although most patients followed a typical maturation pattern, we also observed occasional persistence of low avidity antibodies at later time points, including one case at T4. Similar atypical maturation profiles have been reported in other infections such as BDV, where individual variability, immune impairment, low antigenic exposure, or assay related factors may result in atypical affinity maturation profiles (Allmang et al., 2001). An additional consideration is the potential impact of antibody cross-reactivity resulting from previous Orthopoxvirus exposure or vaccination, which may influence IgG avidity measurements. This aspect is particularly relevant given the increasing use of MVA-BN vaccination. Although none of the participants in our cohort had a documented history of prior Orthopoxvirus exposure or vaccination, including MVA-BN, this potential effect should be further investigated in future studies evaluating avidity-based assays. Another important observation is the inverse correlation observed between IgG avidity and neutralizing antibody titers. This observation is consistent with the temporal dynamics of the immune response, with early-phase samples characterized by high neutralizing titers and low avidity values, followed by progressive affinity maturation over time. Since nAb titers are also influenced by the magnitude of the MPXV-specific IgG response, the progressive decline in antibody titers during follow-up may have contributed to the observed inverse association. Similar observations have been reported in other viral infections, where avidity and neutralizing activity capture distinct yet temporally interconnected aspects of the antibody response (Tsai et al., 2013; Harrington et al., 2021). These findings support the concept that avidity testing should be considered a complementary, rather than substitutive, tool for functional serological assessment during follow-up. The longitudinal design, with sampling extending up to one year after symptom onset, strengthened the relevance of our study by enabling a detailed reconstruction of IgG avidity maturation in natural mpox infection, a process that remains largely unexplored. The iIFA-based avidity assay, incorporating a urea dissociation step, represents a flexible approach. It may be particularly useful for emerging infectious diseases lacking standardized commercial assays. Moreover, the maturation pattern observed, characterized by low avidity during early infection followed by a progressive increase over time, is consistent with affinity maturation kinetics reported across other systems.

Nevertheless, some limitations should be acknowledged. The cohort size is relatively small and demographically homogeneous, comprising only men, which may limit the generalizability of the findings. Although the ROC analysis provides useful preliminary information regarding a potential IgG avidity index cutoff, the results obtained and the proposed threshold require validation in larger, independent, and more diverse cohorts. However, the longitudinal design, with repeated sampling over a one-year follow-up, enabled a detailed characterization of the temporal dynamics of IgG avidity maturation.

The iIFA-based method is inherently operator-dependent; variability in fluorescence intensity and slide interpretation may affect reproducibility and preclude high-throughput application (Pereira et al., 2001; Billich et al., 2002; Luciani et al., 2019). In addition, avidity testing for MPXV lacks standardized chaotropic concentrations, incubation times, and validated cutoffs; similar methodological variability has been reported in previous studies and may influence assay performance. Moreover, iIFA-based method, which uses infected cells as the antigen, cannot distinguish between mature virion (MV) and extracellular enveloped virion (EEV).

Finally, as the avidity index is calculated based on the reduction in antibody titer after chaotropic treatment using an endpoint dilution assay, this approach is difficult to apply to low IgG titers; therefore, only sera with IgG titers ≥1:80 were included in the analysis. It should be noted that the iIFA assay used in this study is based on MPXV-infected cells and may be subject to cross-reactivity with antibodies elicited by other Orthopoxvirus exposures, including prior smallpox vaccination, reflecting the high degree of antigenic similarity within the genus. However, all samples were obtained from PCR-confirmed mpox cases, supporting the interpretation of the serological findings in this clinical context.

In summary, our results contribute to addressing an important gap in current knowledge. Whereas prior studies have primarily focused on avidity profiles induced by MVA-BN vaccination, often reporting low and short-lived avidity in naïve individuals (Oom et al., 2025), our findings suggest that natural MPXV infection in naïve individuals leads to substantial and sustained avidity maturation in the majority of patients over the first-year post infection. This distinction between infection-induced and vaccine-induced antibody quality may have implications for sero-surveillance and for interpreting immune status in populations with mixed exposure histories. From a clinical perspective, avidity assessment may provide additional information in specific contexts, such as vaccination strategies. In populations with uncertain infection history, high-avidity antibodies could suggest prior natural exposure, potentially supporting individualized decisions regarding vaccination or booster administration. Conversely, detection of low-avidity antibodies in seropositive individuals might indicate recent infection or suboptimal immune maturation, contexts in which vaccination strategies could differ. Prior (historical) smallpox vaccination has been associated with durable, high-avidity MPXV-specific antibodies, consistent with the notion that pre-existing orthopoxvirus memory behaves like a remote past exposure and drives higher avidity at later time points. Moreover, although the correlation between avidity and neutralizing activity appears to be influenced by infection timing, the AI can provide valuable temporal information and may complement established serological tools aimed at distinguishing recent from past infection. Similar patterns have been described in other viral infections, including SARS-CoV-2, where avidity has been reported to show an inverse relationship with neutralizing responses at specific stages of the immune response (Monroe et al., 2022; Löfström et al., 2025). These observations suggest that avidity and neutralizing activity capture related but distinct aspects of the humoral response: avidity reflects affinity maturation over time, whereas neutralizing antibodies are indicative of functional antiviral activity that may fluctuate according to antigen exposure and immune waning.

From a diagnostic perspective, IgG avidity may represent a complementary parameter to improve the interpretation of MPXV serology based on IgM and IgG detection, particularly in distinguishing recent from past infection in individuals with unclear exposure history or in epidemiological settings. However, given the exploratory nature of this longitudinal study, further research are needed to confirm our findings, and their potential clinical applicability requires validation in larger and more diverse cohorts, as well as the use of higher-throughput methodologies.

## Data Availability

The datasets presented in this article are not readily available because The data supporting the findings are available from the corresponding author upon reasonable request, but only for sections that do not contain personal information. Requests to access the datasets should be directed to francesca.colavita@inmi.it.
